# Can cardiac iodine-123 metaiodobenzylguanidine imaging contribute to risk stratification in heart failure patients?

**DOI:** 10.1007/s00259-008-0722-4

**Published:** 2008-01-22

**Authors:** Jeroen J. Bax, Mark Boogers, Maureen M. Henneman

**Affiliations:** Department of Cardiology, Leiden University Medical Center, Albinusdreef 2, 2333 ZA Leiden, The Netherlands

Chronic heart failure represents a major challenge in clinical cardiology in terms of the number of patients involved. Over the past years, the number of heart failure patients has increased exponentially, with recent estimations indicating that five million patients in the United States have chronic heart failure, with 550,000 new patients diagnosed annually, resulting in one million hospitalizations [1]. The diagnostic and therapeutic costs involved with heart failure are estimated to be more than $ 29 billion per year [1]. The major part of costs appears related to hospitalizations for decompensated heart failure.

The main cause of heart failure is chronic coronary artery disease. Gheorghiade and Bonow [2] pooled 13 randomized, multicenter heart failure drug trials (with more than 20,000 patients) published in the New England Journal of Medicine between 1986 and 1997. It was concluded that coronary artery disease was the underlying etiology in almost 70% of the patients, and this figure is probably higher because many patients in the trials did not undergo coronary angiography.

Long-term prognosis of heart failure patients remains poor, despite advances in different therapies. Recent data from the Framingham study demonstrated a 5-year mortality rate of 59% for men and 45% for women with heart failure [3]. To improve survival, adequate risk stratification is needed.

The neurohormonal system (adrenergic nervous system and renin-angiotensin aldosteron system) plays a major role in the pathophysiology of heart failure. One of the characteristics is hyperactivity of the sympathetic nervous system with an increase in plasma norepinephrine; at first, this increased activity is supporting the cardiovascular system by increasing heart rate, contractility, and venous return. In chronic heart failure, however, this hyperactivity is unfavorable and may result in desensitization and downregulation of myocardial ß-adrenoceptor with further impairment of cardiac performance and poor outcome.

Accordingly, assessment of the sympathetic nerve activity and cardiac denervation may provide prognostic information in patients with heart failure. Noninvasive scintigraphic visualization of cardiac sympathetic nervous system can be performed by positron emission tomography (PET) and single photon emission computed tomography (SPECT). The most frequently evaluated PET tracer is C11-hydroxyephedrine (HED). In heart failure patients, global sympathetic denervation has been demonstrated with HED PET [4, 5]. Moreover, the reduction in HED uptake was associated with reduced left ventricular (LV) function, indicating a link between altered innervation and LV function [6]. Most important, Pietila et al. [7] reported on the prognostic value of reduced HED uptake in 46 heart failure patients. Over a mean follow-up period of 55 ± 19 months, 11 cardiac deaths occurred and two underwent heart transplantation. Multivariate analysis demonstrated that peak oxygen uptake, LV end-diastolic volume, and HED retention were the only predictors of outcome.

The majority of clinical innervation studies, however, have been performed with iodine-123 metaiodobenzylguanidine (MIBG) [8, 9] and planar or SPECT imaging. This tracer is a norepinephrine analog, which is taken up and stored in the myocardium similarly to norepinephrine [10] but does not undergo further metabolism and is retained in sympathetic nerve endings, providing a strong signal for imaging. The vast majority of MIBG studies have used planar imaging, only the more recent studies have used SPECT imaging [8, 9]. The imaging protocol usually includes acquisition of an early and late dataset; the early images are obtained 10–20 min after tracer injection, whereas the late images are obtained 3–4 h later. The imaging parameters obtained with planar imaging include the early and late heart-to-mediastinum (H/M) ratio and the washout rate, and all provide information on *global* cardiac sympathetic innervation and denervation. With SPECT imaging, it is also possible to evaluate *regional* innervation or denervation.

Various studies have published on the use of MIBG imaging in relation to prognosis in heart failure patients [8, 9]. Most of these studies employed the late H/M ratio as prognostic marker. Merlet et al. [11] evaluated 90 patients with ischemic and nonischemic heart failure; these patients underwent planar MIBG imaging (images obtained 4 h after tracer injection) in addition to routine care (chest X-ray, echocardiography, and radionuclide LV ejection fraction (LVEF) assessment). During a follow-up period of maximum 27 months, ten patients underwent cardiac transplantation, and 22 died. Among all clinical and imaging variables, the H/M ratio on MIBG imaging was the only predictor of event-free survival.

The same group [12] subsequently evaluated 112 patients with heart failure and dilated cardiomyopathy (NYHA class II–IV, LVEF <40%, LV end-diastolic diameter 70 ± 8 mm, and pulmonary capillary wedge pressure 19 ± 8 mm Hg) who underwent planar MIBG imaging 4 h after tracer injection. Among all variables, only the H/M ratio on MIBG imaging and the LVEF were predictive for long-term survival (mean follow-up 27 ± 20 months). Of note, Merlet et al. [11, 12] used a cutoff value for the H/M ratio of 1.2 to identify reduced MIBG uptake. Cohen-Solal and coworkers [13] evaluated 93 heart failure patients with early and late planar MIBG imaging and demonstrated that the reduction in H/M ratio on the delayed MIBG images was related to LVEF, cardiac index, pulmonary wedge pressure, and peak oxygen uptake. Moreover, the H/M ratio (cutoff value 1.2) and peak oxygen uptake were predictive of death or cardiac transplantation over 10 ± 8 months follow-up.

Nakata et al. [14] evaluated 414 patients, with 173 having symptomatic heart failure; all underwent early (10 min) and late (3–4 h) planar MIBG imaging. Over a mean follow-up of 22 ± 7 months, 37 cardiac deaths occurred. Five main predictors of death were identified including an H/M ratio on late MIBG imaging, LVEF ÿ%, NYHA class III or IV, age >60 years and a history of myocardial infarction. Of note, Nakata et al. [14] used a cutoff value of 1.74 to define a reduced H/M ratio.

Wakabayashi et al. [15] compared the prognostic value of cardiac MIBG imaging in patients with ischemic (*n* = 76) and nonischemic cardiomyopathy (*n* = 56). Over a maximum period of 55 months, 69 cardiac deaths occurred. Both in the patients with ischemic and nonischemic cardiomyopathy, the late H/M ratio was the strongest predictor of survival. Importantly, the cutoff values for H/M ratios were 1.5 in ischemic patients, and 2.0 in nonischemic patients.

In addition to the late H/M ratio, the prognostic value of other MIBG parameters has also been reported. Ogita et al. [16] evaluated 79 heart failure patients with LVEF <40% with planar MIBG imaging; both early (20 min) and late (3–4 h) images were obtained. A washout rate ÿ% was a strong predictor of survival. Yamada et al. [17] also demonstrated that washout rate (cutoff value 27%) was the strongest predictor of survival in 65 heart failure patients with LVEF <40%, over a mean follow-up period of 34 ± 19 months. Anastasiou-Nana et al. [18] evaluated 52 heart failure patients with LVEF <40%, and demonstrated that the early H/M ratio was the best predictor for long-term (2-year) outcome. Of note, the early H/M ratio was obtained at 1 h after tracer injection, whereas most studies used a 10- to 20-min interval to obtain the early images.

Only few studies performed a thorough comparison between the different MIBG parameters to predict long-term outcome. Wakabayashi et al. [15] demonstrated in 132 patients that the late H/M ratio provided superior prognostic information over the early H/M ratio and the washout rate. Cohen-Solal et al. [13] reported in 93 heart failure patients that the late H/M ratio provided prognostic information whereas the early H/M ratio and washout rate did not. Although currently available data suggest that the late H/M ratio may be the best prognostic parameter that can be obtained from planar MIBG imaging, it is clear that further studies are needed to solve this issue.

In addition, the optimal cutoff value for the H/M ratio on late MIBG imaging is unclear, and various cutoff values have been used. This is (at least partially) related to the lack of standardization of data acquisition and data analysis. Accordingly, larger studies are needed to obtain these optimal cutoff values. In this perspective, the study by Agostini et al. [19] in the current issue of the journal is a very important contribution. In this analysis, data from 290 heart failure patients (121 ischemic and 169 nonischemic cardiomyopathy, obtained in six European centers) who underwent planar MIBG imaging are re-analyzed in a core laboratory. In this core laboratory, H/M ratios were obtained from the digitized images; only late H/M ratios were derived because early MIBG scans were not available in 72% of patients. The late images were obtained between 2 and 7 h after tracer injection. All patients had a predefined 2-year follow-up period, and patients with less than 2-year follow-up were excluded. Over this period, a total of 67 patients (23% of the population) experienced an event, including 18 cardiac deaths, 44 cardiac transplantations, and five potentially lethal ventricular arrhythmias. The mean H/M ratio was significantly different between patients with and without events (1.51 versus 1.97, *P* < 0.001). Based on receiver operating characteristic (ROC) curve analysis, a cutoff value for H/M ratio of 1.75 yielded a sensitivity of 84% with a specificity of 60% to predict events. Based on this cutoff value, the 2-year event-free survival was 92% for patients with an H/M ratio ÿ.75 as compared to 62% for patients with an H/M ratio <1.75 (*P* < 0.001). When the H/M ratios were divided into quartiles, the 2-year event-rate in the lowest quartile (ÿ.45) was almost 50%, as compared to 1.4% in the highest quartile (ÿ.18). These data provide further support for the use of the late H/M ratio for prognostic purposes in heart failure patients, with a proposed cutoff value of 1.75 for the late H/M ratio. Although the current study is a major step forward in the direction of clinical use of MIBG imaging in heart failure patients, several limitations are important. First, the retrospective nature and the non-uniformity in data collection (different timings for acquisition of the late H/M ratio, ranging from 2 to 7 h after tracer injection, with different dosages of MIBG, different gamma cameras, and different collimators) should be acknowledged. Furthermore, a substantial percentage of patients had mild heart failure, with 57% in NYHA class II and 34% of patients having an LVEF >35%. It is anticipated that patients with more severe heart failure have lower H/M ratios and the optimal cutoff value for prediction of events will be lower than 1.75; although a sub-analysis of patients with LVEF ÿ% is included, an ROC curve analysis for these patients is not presented. Despite these shortcomings, the study by Agostini et al. [18] adds substantially to the existing literature and provides further evidence supporting the use of MIBG in risk stratification of heart failure patients.
